# 文物中多糖类胶结材料的热裂解-气相色谱/质谱识别

**DOI:** 10.3724/SP.J.1123.2022.03005

**Published:** 2022-08-08

**Authors:** Na WANG, An GU, Yajie QU, Yong LEI

**Affiliations:** 故宫博物院, 书画保护文化和旅游部重点实验室, 北京 100009; The Palace Museum, Key Laboratory of Calligraphies and Paintings Conservation (The Palace Museum), Ministry of Culture and Tourism, Beijing 100009, China

**Keywords:** 热裂解-气相色谱/质谱, 多糖, 胶结材料, 淀粉, 桃胶, 阿拉伯胶, pyrolysis-gas chromatography/mass spectrometry (Py-GC/MS), polysaccharides, binding material, starch, peach gum, gum Arabic

## Abstract

热裂解-气相色谱/质谱(Py-GC/MS)技术能够实现微量样品中有机组分的准确、快速检测,非常适用于文物中各类天然有机材料的定性分析。该研究以中国古代书画、建筑、器物等文化遗产中常用的淀粉、桃胶,以及西方文化遗产中常用的阿拉伯胶等多糖类胶结材料为研究对象,系统分析并总结各类材料的Py-GC/MS特征裂解组分及辨别方法。研究发现,淀粉、桃胶、阿拉伯胶在色谱保留时间前段的裂解产物基本一致,主要是小分子呋喃、酮类组分;在保留时间中段3类材料的裂解产物主要是呋喃型酮等组分,但不同材料的具体裂解组分差异明显;在保留时间后段,3类材料检出多种单糖衍生物以及单糖低聚体衍生物,其中桃胶与阿拉伯胶裂解组分较为接近,但与淀粉完全不同。因此,可根据不同保留时间段淀粉、桃胶、阿拉伯胶裂解产物的差异实现3类材料的辨别,其中1,6-脱水-*β*-D-吡喃葡萄糖只在淀粉中检出且色谱峰强度高,可以作为识别淀粉的特征组分;此外,可根据桃胶、阿拉伯胶在保留时间后段的裂解产物主要质谱碎片离子*m/z* 60、*m/z* 101的提取离子流图分布特征实现其辨别。基于所建立的Py-GC/MS方法,研究推断故宫旧藏清代剔红云龙纹天球瓶瓶口部位黏结材料含有面粉,旻宁御笔并蒂含芳贴落画心纸所用黏结材料为面粉糨糊。该研究所建立方法及所总结的数据易于推广,适用于我国文物中多糖类材料的准确、快速识别,研究结果能为相关文物材质工艺的研究以及保护修复方案的制定提供科学依据。

## Abstract (en)

Pyrolysis-gas chromatography/mass spectrometry (Py-GC/MS) is suitable for the qualitative analysis of natural organic materials in cultural relics owing to its ability of accurately and rapidly identifying trace quantities of organic components in samples. In the present study, pyrolytic components of starch and peach gum, which are commonly used in ancient Chinese cultural relics such as calligraphic works, paintings, architecture, and objects, as well as gum Arabic, which is commonly used in western cultural relics, were systematically characterized using Py-GC/MS. As polysaccharide materials are often found mixed with other organic materials in cultural relics, an online methylation Py-GC/MS procedure previously established by the authors’ research group, which is suitable for the qualitative analysis of drying oils, proteins, and waxes, was performed to analyze the starch, peach gum, and gum Arabic reference samples. The established experimental procedure can be used to comprehensively analyze the polysaccharides and other organic materials in cultural relics owing to its slow heating rate, long running time, wide mass spectrometry detection range, and high chromatographic separation efficiency.

The experimental results were examined by dividing the pyrolytic products into three sections depending on the retention time. In the first section (2.5-10 min), pyrolytic products from the three sample types were similar and mostly included small molecule furans and ketones. However, the peach gum and gum Arabic contain a wider variety of pyrolytic products than starch, possibly because starch contains only glucose, whereas peach gum and gum Arabic contain a variety of monosaccharides. In the middle section (10-19 min), the pyrolytic products of starch, peach gum, and gum Arabic mostly consisted of furanones and other components. However, a unique profile was produced for each material type. Nevertheless, it should be noted that 2-methoxy-phenol and maltol were detected in all three materials with high chromatographic peak intensities. Therefore, these components are suitable markers for the identification of polysaccharides. In the last section (19-60 min), a variety of monosaccharide derivatives and monosaccharide oligomer derivatives were detected in the reference samples. The pyrolytic products of peach gum and gum Arabic were similar but completely different from those of starch. Therefore, starch, peach gum, and gum Arabic could be distinguished according to their pyrolytic products. In particular, 1,6-anhydro-*β*-D-glucopyranose was detected in starch with an extremely high intensity and was undetected in either of the other reference materials. Thus, this compound could act as a characteristic pyrolytic component for the identification of starch. Peach gum and gum Arabic could be rapidly distinguished according to the extracted ion chromatograms for *m/z* 60 and *m/z* 101, which represented major fragments of the pyrolytic products of peach gum and gum Arabic in the last section of the chromatogram.

The established Py-GC/MS method was successfully applied to the identification of starch in binding materials from the rim of a globular carved red lacquer vase with dragon and cloud motifs, as well as in paper collected from a *Tieluo* with Bingdihanfang written by Min-ning. Both objects are part of the Palace Museum collection and originate from the Qing Dynasty. Research results of the present work are easy to be popularized. This study provides a method suitable for the accurate and rapid identification of polysaccharide materials in cultural relics, as well as a scientific basis for the research, conservation, and restoration of similar cultural relics. However, it should be noted that aging and the presence of other organic or inorganic materials in cultural relics may interfere with the detection of polysaccharide materials. Therefore, a further investigation on the aging behavior of polysaccharides and the effects of other materials on the identification of polysaccharides is required.

多糖类材料,包括淀粉、植物胶等,是我国文物制作及修复过程中常用的胶结材料,如淀粉常与桐油、猪血等混合用作古建地仗的胶结材料^[1,2]^,淀粉糨糊是书画、档案、裱作所用传统修裱材料^[3⇓-5]^,而桃胶则可在陶器修补、壁画揭取工作中起加固作用^[6,7]^。

所用材料的准确识别,是对文物进行科学研究及合理制定修复、保护方案的基础。多糖类材料属于碳水化合物,是由不少于10个的单糖通过糖苷键聚合而成的高分子聚合物,原始成分复杂,此外,多糖在文物制作及修复过程中常与其他有机、无机材料混用,且所用量一般较少,如古建地仗中用来胶结灰料的油满,其所用面粉、石灰水、灰油的质量比约为1∶1.3∶1.95,面粉用量最少^[1]^,因此文物样品中多糖类材料的检测较为困难。

考古工作者通常通过显微观测的方法,来确定历史遗存中是否含有淀粉颗粒并判断其种属,目前已有多类植物的淀粉粒形态数据库^[8,9]^,此类方法也可用于淀粉类胶料的定性分析^[10]^,但无法直接用于植物胶类材料的检测,而且显微观测方法往往需要耗费较长时间对样品进行浸泡、提取等前处理,且所需样品量较多,因此并不适于快速判断微量样品中是否含有淀粉胶料。傅里叶变换红外光谱(FT-IR)分析操作简单,可快速辨别样品中是否含有多糖类材料,但在文物样品检测中谱图解析易受其他组分干扰,且FT-IR分析无法实现淀粉、植物胶等多糖的具体辨别。气相色谱/质谱(GC/MS)^[11⇓-13]^、高效液相色谱(HPLC)^[14,15]^技术可通过对多糖单体的定性、定量识别来判断其类别,但GC/MS、HPLC实验需对样品进行水解、衍生化等前处理,过程繁复、耗时长,且对于文物样品来说,前处理过程有可能造成目标组分的流失。

在GC/MS技术基础之上发展起来的热裂解-气相色谱/质谱(Py-GC/MS),可在高温及惰性气体保护下将待测样品中的有机高分子材料瞬间热裂解为小分子组分,裂解组分随即通过GC/MS系统分离并识别,最终可根据裂解产物的组成还原出样品原始组分信息。Py-GC/MS实验无需样品前处理,能避免前处理过程中待测组分的流失,而且兼具GC/MS高灵敏度的特点,能实现微量、复杂样品的定性分析,因此,Py-GC/MS技术已被广泛应用于文物样品中各类天然及合成有机高分子材料的综合分析^[16,17]^,本课题组已基于此项技术,建立了大漆^[18]^、干性油^[19]^、蛋白质^[20]^以及蜡类材料^[21]^的定性识别方法。

目前已有科研工作者将Py-GC/MS技术用于多糖类材料的识别及研究^[16,22,23]^,但鲜见有关文物用多糖类胶结材料Py-GC/MS分析结果的详细报道及不同类别多糖辨别方法。我国最常用的多糖类胶结材料是淀粉及桃胶,而阿拉伯胶则在西方国家被广泛使用^[24]^,鉴于此,本研究将以小麦淀粉糨糊、桃胶、阿拉伯胶为研究对象,系统总结各类材料的Py-GC/MS特征裂解产物及辨别信息,以实现这3种材料的准确、快速辨别。在参比材料研究的基础上,将所建立分析方法应用于故宫旧藏剔红云龙纹天球瓶瓶口部位黏结材料,以及旻宁御笔并蒂含芳贴落画心纸所用黏结材料的辨别,以验证所建立分析方法的可行性。

## 1 实验部分

### 1.1 仪器、试剂与材料

Py-GC/MS分析采用日本Frontier公司EGA-PY3030D型热裂解仪,美国Agilent公司7890B/5977A型气相色谱/质谱联用仪。

淀粉、天然桃胶均由故宫博物院文保科技部修复科组提供,阿拉伯胶购于Kremer试剂公司。淀粉为小麦淀粉,制成糨糊后用平头画笔均匀刷涂于玻璃板上,放置在室温条件下待干燥至恒重后即可进行分析。桃胶、阿拉伯胶直接取样测试。

甲基化试剂为10%(质量分数)四甲基氢氧化铵(tetramethylammonium hydroxide, TMAH)甲醇溶液,购于上海阿拉丁试剂公司,分析纯。

### 1.2 实验条件

实验采取在线甲基化技术,将少于1 mg的样品与5 μL 10% TMAH甲醇溶液一同放入不锈钢样品舟,然后直接送入热裂解仪石英裂解管,样品甲基化反应可与热裂解反应同时完成。裂解温度500 ℃,裂解时间为12 s。热裂解仪与气相色谱接口温度为300 ℃。

GC条件 色谱柱为HP-5MS毛细管柱(30 m×0.25 mm×0.25 μm);进样口温度为300 ℃;色谱柱初始温度50 ℃,保持2 min,随后柱温箱以4 ℃/min上升到300 ℃,保持15.5 min;分流进样,分流比50∶1;载气为氦气,流速1.0 mL/min。

质谱条件 电子轰击离子源温度230 ℃,电离源能量70 eV,四极杆温度150 ℃,采取全扫描的模式,扫描范围为*m/z* 29~550,质谱识别数据库为NIST Libraries。

为保证实验结果的准确性,参比样品在相同条件下均重复进行3次Py-GC/MS测试,取其平均值作为最终结果。

### 1.3 文物样品信息

为评价所建立多糖材料分析方法及参数的有效性,对两个采集自故宫旧藏清代文物的样品进行分析,以识别其所用胶结材料。两个样品分别为剔红云龙纹天球瓶(以下简称剔红瓶)瓶口部位黏结材料,以及旻宁御笔并蒂含芳贴落(以下简称贴落)画心纸,文物照片见图1。

**图1:**
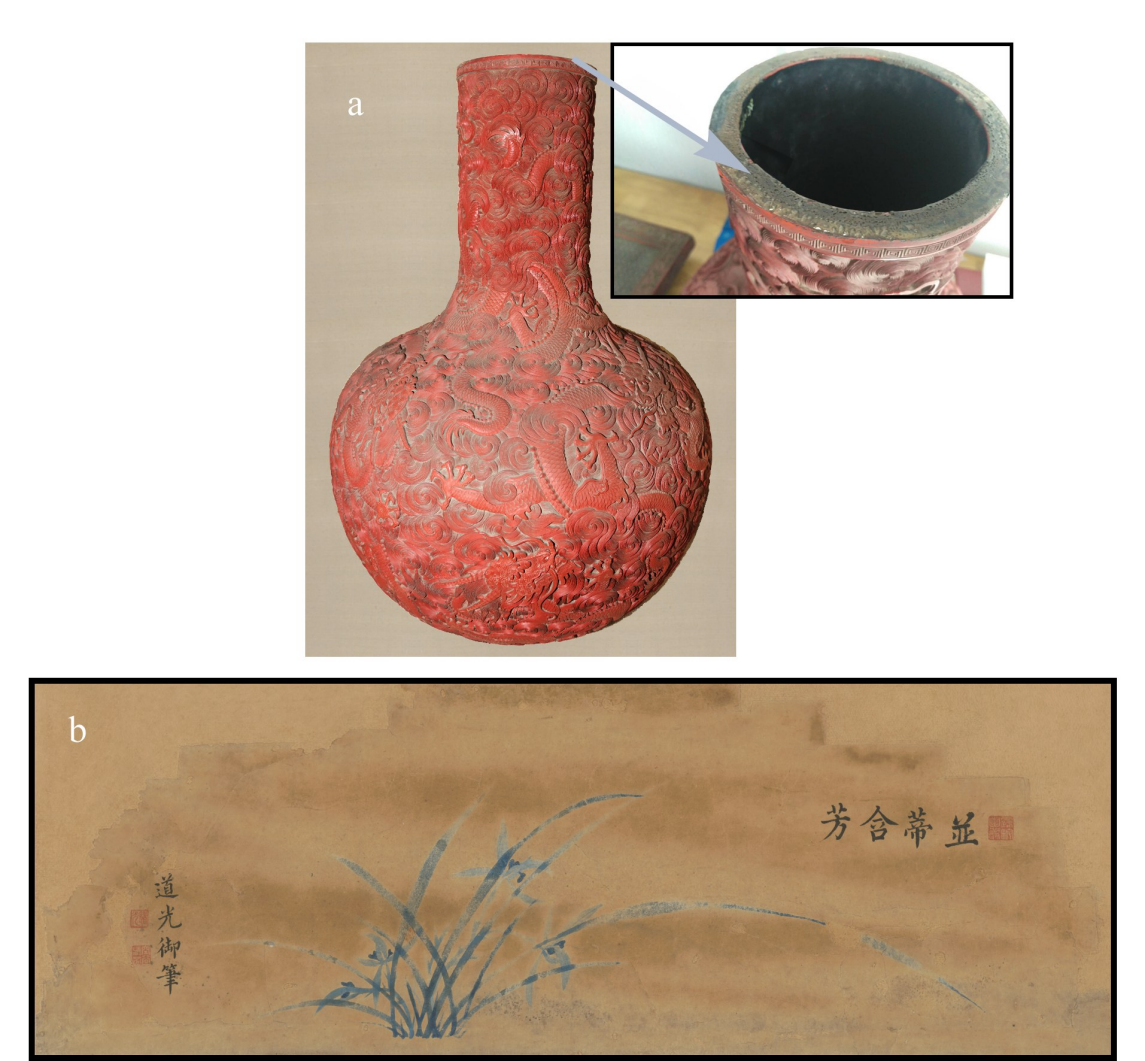
故宫博物院旧藏清代(a)剔红云龙纹天球瓶及其 瓶口部位黏结材料和(b)旻宁御笔并蒂含芳贴落

## 2 结果与讨论

### 2.1 Py-GC/MS分析条件

由于文物样品中多糖类胶结材料常与其他有机材料混用,因此研究采用课题组已建立的同时适用于干性油^[19]^、蛋白质^[20]^、蜡类材料^[21]^定性分析的在线甲基化Py-GC/MS实验程序(即本文1.2节所述实验参数)对淀粉、桃胶、阿拉伯胶等多糖胶结材料标准样品进行分析。已建立的Py-GC/MS程序柱温箱升温速率缓慢、运行时间长、质谱检测范围大,能够实现文物样品中多糖及其他类别有机材料的综合分析。此外,在线甲基化反应的进行,能够将样品中的极性组分甲基化,起到改善色谱峰形、提高色谱柱分离效率的作用。

### 2.2 多糖类胶结材料参比样品Py-GC/MS分析

表1所列为淀粉、桃胶、阿拉伯胶所含单糖及糖醛酸的具体组分。其中淀粉是由D-葡萄糖脱去水分子后经糖苷键连接在一起形成的同多糖,而桃胶、阿拉伯胶则是由阿拉伯糖、半乳糖、葡糖糖醛酸等多种不同单糖以及糖醛酸组成的杂多糖^[25⇓-27]^。

**表1:** 淀粉、桃胶、阿拉伯胶所含单糖和糖醛酸的组成

Sample	Glucose	Arabinose	Rhamnose	Galactose	Xylose	Mannose	Uronic acid
Starch^[25]^	√						
Peach gum^[26]^		√	√	√	√	√	√
Gum Arabic^[27]^		√	√	√			

√ indicates the presence of component.

淀粉、桃胶、阿拉伯胶的Py-GC/MS分析结果总离子流图(TIC)见图2。经谱图解析,发现在不同保留时间段内,淀粉、桃胶、阿拉伯胶的裂解产物比对结果具有明显差异。表2、表3、表4及表5所列即分别为保留时间前段(2.5~10 min)、中段(10~19 min)、后段(19~60 min)3类材料的主要裂解产物。

**图2:**
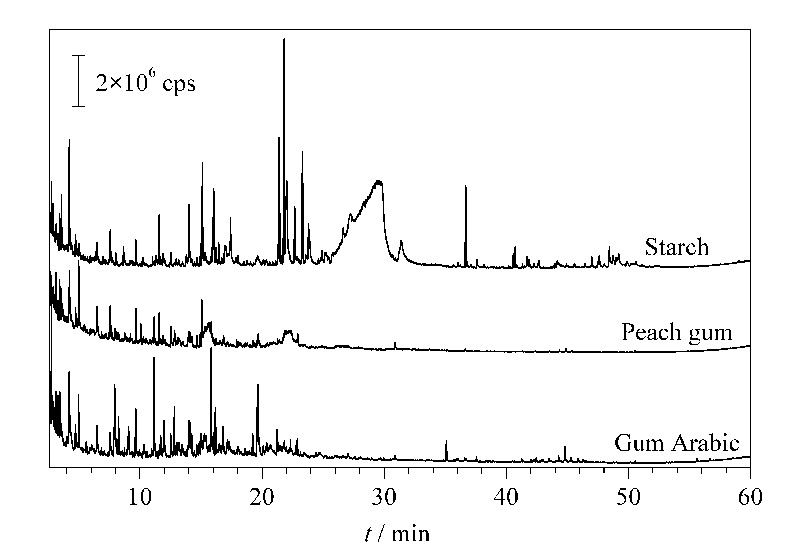
淀粉、桃胶、阿拉伯胶的Py-GC/MS总离子流色谱图

**表2:** 淀粉、桃胶、阿拉伯胶参比样品在保留时间2.5~10 min内的Py-GC/MS分析结果

t_R_/min	Pyrolytic component	Starch	Peach gum	Gum Arabic
2.9	3-methyl-furan	√	√	√
3.1	1-hydroxy-2-butanone		√	√
3.2	1,2-ethanediol monoacetate		√	√
3.3	succindialdehyde		√	√
3.5	1-deoxy-2,4-methylene-3,5-anhydro-D-xylitol			√
3.9	3-furaldehyde	√	√	
4.2	2-(methoxymethyl)-furan			√
4.2	2-cyclopenten-1-one			√
4.3	furfural	√	√	√
4.7	2-butanone		√	√
4.8	2-furanmethanol	√	√	√
5.0	1-(acetyloxy)-2-propanone	√	√	√
5.4	cyclopent-4-ene-1,3-dione	√	√	√
5.6	2,5-dihydro-3,5-dimethyl-2-furanone			√
6.0	2-methyl-2-cyclopenten-1-one		√	√
6.1	1-(2-furanyl)-ethanone	√	√	√
6.2	2(5H)-furanone	√	√	√
6.5	1,2-cyclopentanedione	√	√	√
6.6	2,5-hexanedione		√	√
6.9	5-methyl-2(5H)-furanone		√	√
7.0	3-methyl-2,5-furandione	√		
7.6	5-methyl-2-furancarboxaldehyde	√	√	√
7.7	1-(acetyloxy)-2-butanone	√	√	√
8.0	methyl 2-furoate		√	√
8.8	2,5-dihydro-3,5-dimethyl-2-furanone		√	√
9.5	2,2-dimethylpropanoic anhydride	√		
9.7	3-methylcyclopentane-1,2-dione	√	√	√

**表3:** 淀粉、桃胶、阿拉伯胶参比样品在保留时间10~19 min内的Py-GC/MS分析结果

Sample	t_R_/min	Pyrolytic component	Sample	t_R_/min	Pyrolytic component
Starch	10.3	2-ethyl-2-hexen-1-ol	Gum Arabic	10.4	5-oxotetrahydrofuran-2-carboxylic acid
	11.6	2,5-dimethylfuran-3,4(2H,5H)-dione		11.7	2-methoxy-phenol
	11.7	2-methoxy-phenol		12.0	3-methyl-1,2-cyclopentanedione
	12.6	maltol		12.6	maltol
	13.8	2,3-dihydro-3,5-dihydroxy-6-methyl-4H-pyran-4-one		13.1	5-acetyldihydro-2(3H)-furanone
	13.9	tetrahydro-2H-pyran-2-one		14.0	1-octen-3-yl-acetate
	15.1	5-(2-propynyloxy)-2-pentanol		14.1	4-methoxycarbonyl-4-butanolide
	15.4	3,5-dihydroxy-2-methyl-4H-pyran-4-one		14.2	1,4-dimethoxy-benzene
	16.1	1,4∶3,6-dianhydro-α-D-glucopyranose		15.0	2,6-anhydro-1,3,4-tri-O-methyl-β-D-
	16.3	2,3-anhydro-D-galactosan			fructofuranose
	16.5	2,3-anhydro-D-mannosan		15.7	5-oxotetrahydrofuran-2-carboxylic
	17.0	5-hydroxymethylfurfural			acid ethyl ester
Peach gum	10.2	4-methyl-5H-furan-2-one		15.8	di(5-methoxy-3-methylpent-2-yl)
	10.5	4,5-dimethyl-1,3-dioxol-2-one			glutaric acid ester
	10.6	3-ethyl-2-hydroxy-2-cyclopenten-1-one		16.2	4-methoxy-2,5-dimethyl-3(2H)-
	11.6	2,5-dimethylfuran-3,4(2H,5H)-dione			furanone
	11.7	2-methoxy-phenol		16.6	2-acetyl-5-methylfuran
	11.9	pentanal		17.1	4-(1-methylethyl)-2-cyclohexen-1-one
	12.0	3-methyl-1,2-cyclopentanedione			
	12.6	maltol			
	13.1	5-acetyldihydro-2(3H)-furanone			
	13.9	dihydro-2H-pyran-2,6(3H)-dione			
	15.1	5-(2-propynyloxy)-2-pentanol			
	15.8	di(5-methoxy-3-methylpent-2-yl) glutaric acid ester			
	16.9	4-methoxycarbonyl-4-butanolide			
	18.1	1-(2,5-dihydroxyphenyl)-ethanone			

**表 4:** 淀粉标准样品在保留时间19~60 min内的Py-GC/MS分析结果

t_R_/min	Pyrolytic component	Typical fragments in mass spectra (m/z^a^)
21.8	β-D-2,6-anhydro-1,3,4-tri-O-methyl-fructofuranose	101, 45, 99, 71, 127, 75, 87, 88
22.0	methyl 6-deoxy-2-O-methyl-β-D-allopyranoside	74, 87, 59, 57, 45
22.7	3,4,6-tri-O-methyl-D-glucose	45, 87, 71, 101, 115, 84
23.3	2-O-methyl-D-mannopyranosa	45, 87, 74, 59, 115, 71, 57, 99
24.6	α-methyl 4-methylmannoside	88, 45, 87, 74, 75, 71
25.2-31.0	1,6-anhydro-β-D-glucopyranose	60, 57, 73
25.7	2,6-di-O-methyl-D-galactopyranose	87, 45, 88, 73, 75, 85
27.2	2,3-di-O-methyl-D-xylopyranose	87, 45, 88, 71, 101
28.3	3,4-di-O-acetyl-D-arabinal	98, 43, 73, 99, 115, 81
48.4	monosaccharide oligomer derivative 1^b^	88, 45, 173, 87, 71, 74, 101, 205, 219
48.7	monosaccharide oligomer derivative 2	87, 101, 74, 73, 59, 173, 127, 115
50.2	monosaccharide oligomer derivative 3	87, 74, 45, 127, 88, 101, 59, 191, 263

a: *m/z* were listed in decreasing order of intensity. b: Numbers represent the order in which components identified.

**表5:** 桃胶、阿拉伯胶标准样品在保留时间19~60 min内的Py-GC/MS分析结果

t_R_/min	Pyrolytic component	Typical fragments in mass spectra (m/z)	Peach gum	Gum Arabic
19.2	1,2,3,4-tetramethylmannose	101, 45, 88, 72, 129	√	√
19.7	2,3-di-O-methyl-D-xylopyranose	87, 101, 45, 115, 74	√	√
21.6-22.8	monosaccharide derivative 1	60, 57, 73, 56, 42	√	
21.8	monosaccharide derivative 2	60, 73, 57, 101, 43, 127		√
22.9	methyl-2,4-di-O-methyl-β-L-arabinopyranoside	101, 74, 87, 59, 45	√	√
26.4	1,2,3,4,5-pentamethoxy cyclopentane	101, 88, 45, 72		√
27.0	2,3,4,5-tetra-O-methyl-D-glucose	101, 88, 45, 73, 71	√	√
35.1	monosaccharide oligomer derivative 4	45, 101, 145, 99, 71, 89, 55, 113		√
37.6	monosaccharide oligomer derivative 5	101, 81, 45, 143, 89		√
41.4	monosaccharide oligomer derivative 6	101, 45, 143, 175, 99		√
42.2	monosaccharide oligomer derivative 7	101, 88, 45, 115, 221, 143		√
42.3	monosaccharide oligomer derivative 8	101, 45, 115, 143, 99		√
42.9	monosaccharide oligomer derivative 9	45, 101, 143, 115, 99, 71, 89, 111, 175	√	√
43.0	monosaccharide oligomer derivative 10	101, 45, 111, 143, 71, 87, 115, 175, 99		√
43.5	monosaccharide oligomer derivative 11	101, 45, 87, 71, 59	√	√
44.1	monosaccharide oligomer derivative 12	101, 45, 143, 71, 99, 115, 89, 175		√
44.3	monosaccharide oligomer derivative 13	101, 143, 45, 74, 175, 99, 115, 89, 187, 261	√	√
44.8	monosaccharide oligomer derivative 14	88, 101, 45, 221, 115, 71	√	√
45.3	monosaccharide oligomer derivative 15	101, 88, 45, 219, 71		√
55.6	monosaccharide oligomer derivative 16	101, 143, 175, 45, 115, 261, 99, 89, 71	√	√
58.1	monosaccharide oligomer derivative 17	101, 88, 143, 45, 71, 111, 99, 115, 160	√	√

在保留时间2.5~10 min内,淀粉、桃胶、阿拉伯胶裂解产物主要是3-甲基-呋喃、呋喃酮、呋喃甲醛以及1-羟基-2-丁酮、1,2-环戊二酮等小分子呋喃、酮类组分(见表2),其中桃胶和阿拉伯胶的裂解产物几乎一致,而淀粉裂解产物的种类明显少于其他两种植物胶,这可能是因为淀粉仅由葡萄糖一种单糖聚合而成,而桃胶和阿拉伯胶的单糖构成则更为多样。

在保留时间10~19 min内,淀粉、桃胶、阿拉伯胶的裂解产物差异明显(见表3)。其中淀粉主要检出2,5-二甲基呋喃-3,4(2*H*,5*H*)-二酮、四氢-2*H*-吡喃-2-酮、1,4∶3,6-二酐-*α*-D-吡喃葡萄糖等多种呋喃型酮、吡喃型酮以及单糖脱水产物;桃胶主要检出多种呋喃型酮以及3-甲基-1,2-环戊二酮等多种酮类组分,吡喃型酮仅检出二氢-2*H*-吡喃-2,6(3*H*)-二酮;阿拉伯胶的裂解产物则更为复杂,包括呋喃型羧酸、酮、呋喃型酮、酯、单糖脱水产物等多类组分,但未检出吡喃型酮,其中保留时间为15.8 min的戊二酸双(5-甲氧基-3-甲基-2-戊醇)甲酯,其色谱峰是阿拉伯胶所有裂解产物中的次强峰,可作为辨别阿拉伯胶的特征组分。此外需指出的是,保留时间为11.7 min的2-甲氧基-苯酚、保留时间为12.6 min的麦芽酚在3种材料中都有检出且色谱峰强度较高,可作为辨别多糖类材料的参考组分。

在保留时间19~60 min内,淀粉、桃胶、阿拉伯胶在31 min之前的裂解产物是葡萄糖、甘露糖等单糖经分子内脱水、开环、甲基化等反应形成的单糖衍生物(monosaccharide derivative),而35 min之后则是单糖低聚体衍生物(monosaccharide oligomer derivative)(见表4、表5)。对于不能通过谱库检索确定分子结构的单糖及单糖低聚体衍生物,表中以单糖衍生物、单糖低聚体衍生物加以数字序号进行标识,为更直观的进行辨别,表中依次列出了所有裂解产物的主要质谱碎片离子。对比表4、表5可看出,虽然桃胶中所检测到裂解产物的数量少于阿拉伯胶,但两者裂解产物的种类基本一致,因此可确定桃胶、阿拉伯胶裂解产物相似度较高,但是淀粉的裂解产物与前两者完全不同。

如图2和表4所示,淀粉在保留时间25.2~31.0 min检出的1,6-脱水-*β*-D-吡喃葡萄糖是其主要裂解产物。1,6-脱水-*β*-D-吡喃葡萄糖的质谱图及其主要碎片离子*m/z* 60、57、73的提取离子流图(EIC)见图3。进一步对比其最强碎片离子*m/z* 60的EIC图在淀粉、桃胶、阿拉伯胶中的分布情况(见图4),可确定1,6-脱水-*β*-D-吡喃葡萄糖仅在淀粉中检出,因此可将1,6-脱水-*β*-D-吡喃葡萄糖作为辨别淀粉与桃胶、阿拉伯胶的特征组分。此外,虽然淀粉是由葡萄糖构成的同多糖,但其裂解产物中还检出了果糖、阿洛糖、甘露糖、半乳糖、木糖等其他单糖的衍生物,这可能是由于淀粉热裂解过程中在高温、高压及碱性条件下发生了分子异构反应,导致葡萄糖结构发生变化。

**图3:**
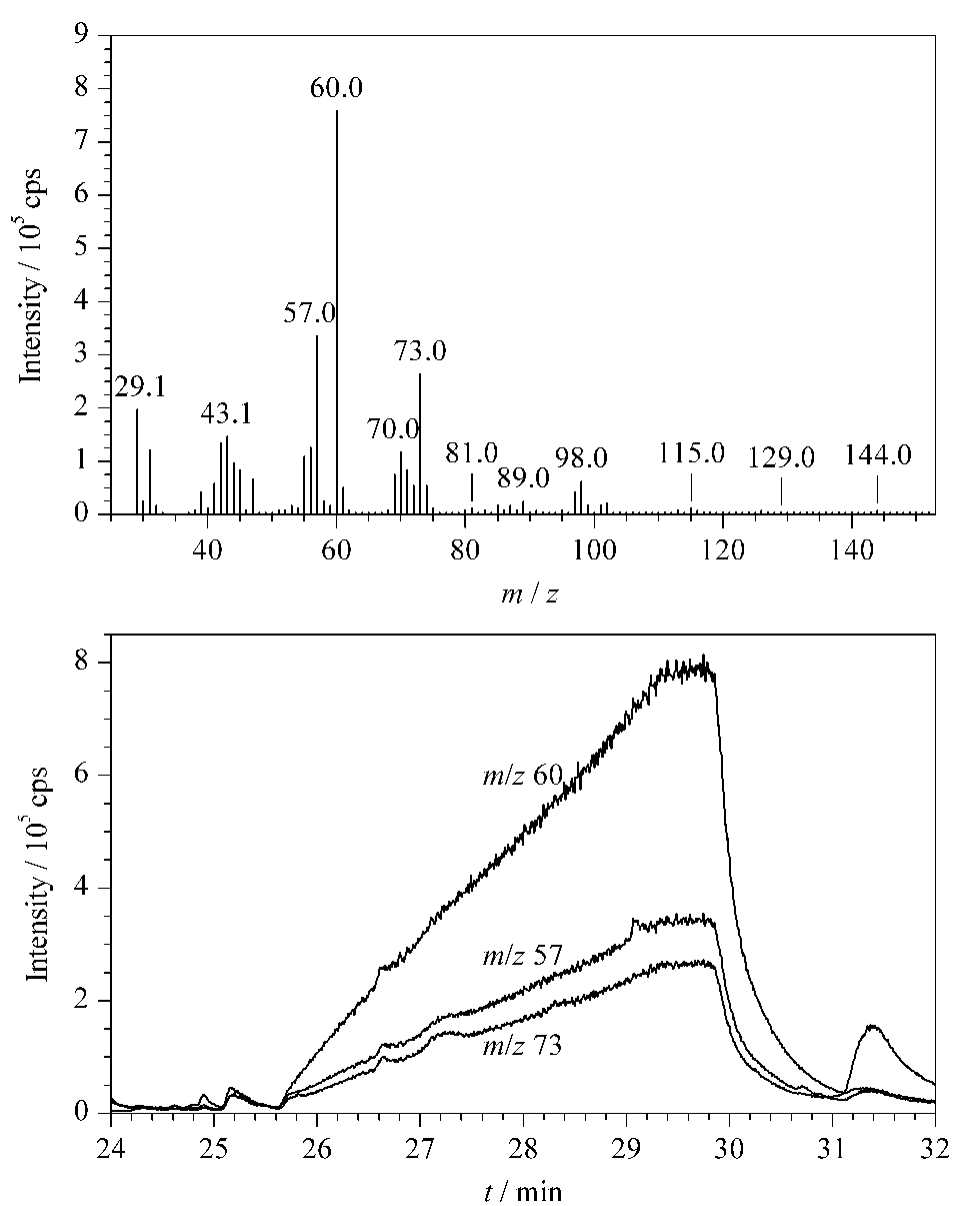
淀粉在保留时间25.2~31.0 min的裂解产物1,6- 脱水-*β*-D-吡喃葡萄糖的质谱图及其主要碎片离子的提取离子流图

**图4:**
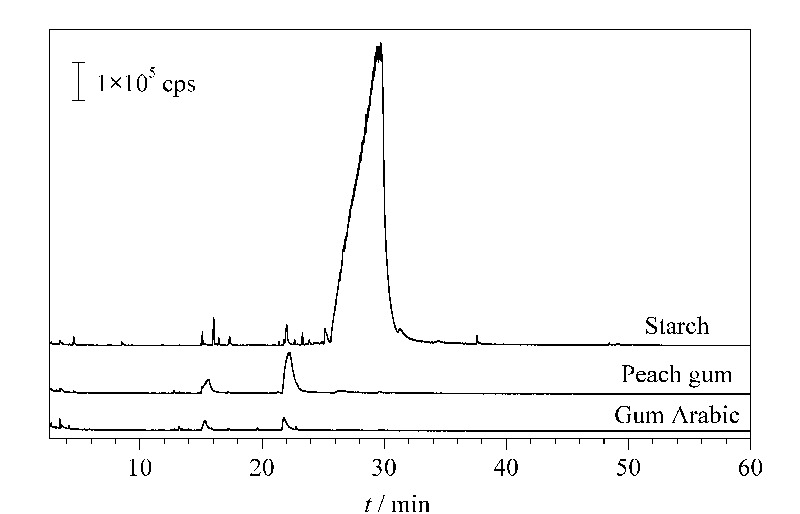
淀粉、桃胶、阿拉伯胶Py-GC/MS分析的*m/z* 60 碎片离子的提取离子流图

根据表5所列桃胶、阿拉伯胶裂解产物的主要质谱碎片离子质荷比,可看出除桃胶在21.6~22.8 min检出的单糖衍生物1、阿拉伯胶在21.8 min检出的单糖衍生物2的最强碎片离子为*m/z* 60之外,所有单糖衍生物及单糖聚体的最强或次强质谱碎片离子都是*m/z* 101。图5所示为桃胶、阿拉伯胶的*m/z* 60、*m/z* 101碎片离子的EIC图,可看出,桃胶在21.6~22.8 min的*m/z* 60的EIC峰强度远高于所有*m/z* 101的EIC峰,而阿拉伯胶在21.8 min的*m/z* 60的EIC峰强度弱于大部分*m/z* 101的EIC峰,因此可以通过*m/z* 60、*m/z* 101的EIC图中峰保留时间及强度对比实现桃胶与阿拉伯胶的辨别。

**图 5:**
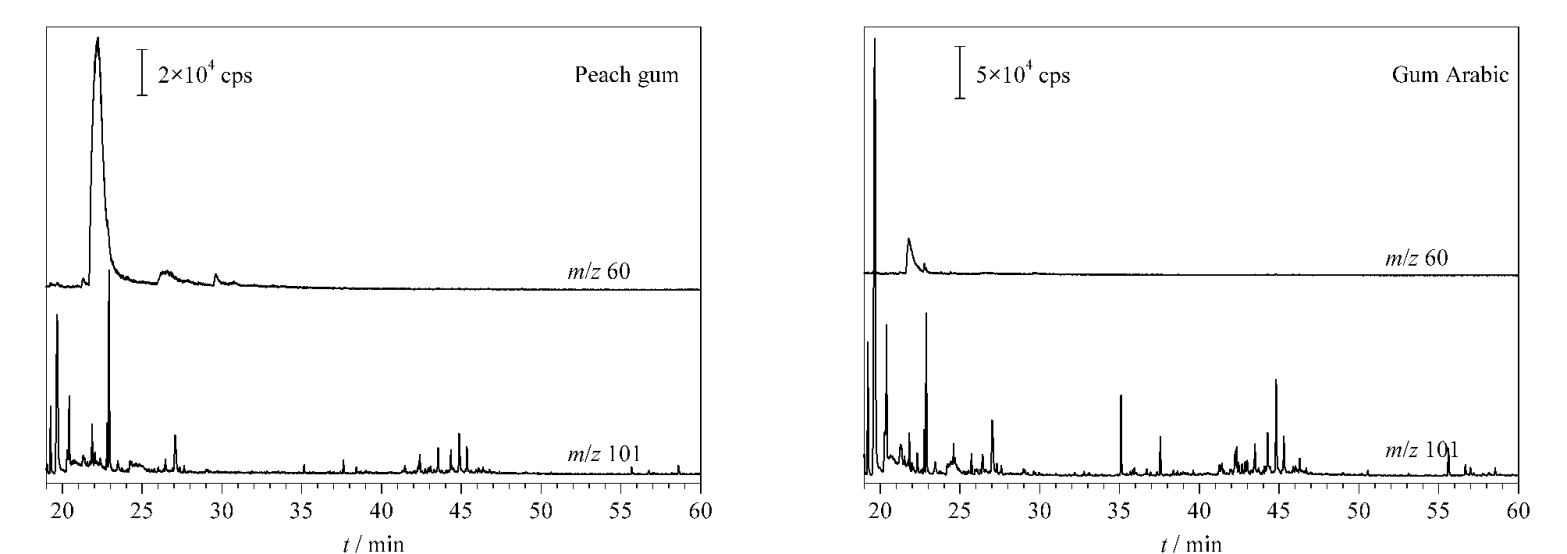
桃胶、阿拉伯胶的Py-GC/MS分析在保留时间19~60 min内*m/z* 60、*m/z* 101碎片离子的提取离子流图

综上所述,淀粉、桃胶、阿拉伯胶在本工作所用色谱分离方法保留时间前段(2.5~10 min)、中段(10~19 min)、后段(19~60 min)内的裂解产物差异明显,特别是可根据中段及后段裂解产物的差异实现3类材料的辨别。

### 2.3 文物样品分析

为评价所建立多糖类材料的Py-GC/MS分析方法及所总结裂解产物信息的可行性,将其应用于故宫旧藏剔红瓶瓶口部位黏结材料以及贴落画心纸所用胶结材料的分析。

考虑到实际文物样品组分复杂,其所含的其他有机、无机材料可能会干扰到多糖裂解产物的识别,因此仔细分析了文物样品Py-GC/MS实验结果中所有色谱峰的质谱图,而不仅限于强度较高的峰,并且为确保辨别结果的准确性,只有当文物样品色谱峰的保留时间以及质谱图均能与多糖标准样品相对应,才认定此组分可归属于多糖裂解产物。

两件文物样品的Py-GC/MS分析结果见图6,经质谱解析,在两个样品中均识别出大量多糖类材料裂解产物(见表6),特别是保留时间25~30 min内1,6-脱水-*β*-D-吡喃葡萄糖的检出可确定两件文物样品中都有淀粉胶结材料,且根据两类文物传统用料特点,推测剔红瓶瓶口部位黏结材料中含有面粉,贴落画心纸同样采用面粉糨糊。

**图 6:**
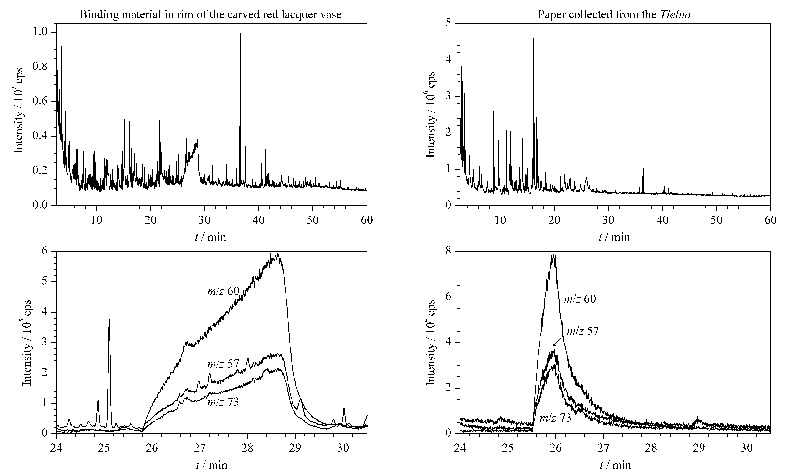
剔红瓶瓶口部位黏结材料和贴落画心纸样品的Py-GC/MS分析结果以及裂解产物1,6-脱水-β-D-吡喃葡萄糖 主要碎片离子m/z 60、m/z 57、m/z 73的EIC图

**表6:** 剔红瓶瓶口部位黏结材料和贴落画心纸样品的Py-GC/MS分析结果

t_R_/min	Pyrolytic component	Binding material in rim of the carved red lacquer vase	Paper collected from the Tieluo
3.0	3-methyl-furan	√	
4.0	3-furaldehyde	√	√
4.3	furfural	√	√
4.7	2-butanone	√	√
4.8	2-furanmethanol	√	
5.0	1-(acetyloxy)-2-propanone	√	
5.4	cyclopent-4-ene-1,3-dione	√	
5.9	2-methyl-2-cyclopenten-1-one		√
6.1	1-(2-furanyl)-ethanone	√	√
6.2	2(5H)-furanone	√	
6.6	1,2-cyclopentanedione	√	√
6.9	5-methyl-2(5H)-furanone	√	
7.5	5-methyl-2-furancarboxaldehyde	√	
7.6	1-(acetyloxy)-2-butanone	√	
9.7	3-methylcyclopentane-1,2-dione	√	√
11.5	2,5-dimethylfuran-3,4(2H,5H)-dione	√	
11.7	2-methoxy-phenol	√	√
12.7	maltol	√	√
16.0	1,4∶3,6-dianhydro-α-D-glucopyranose	√	√
16.3	2,3-anhydro-D-galactosan	√	
16.5	2,3-anhydro-D-mannosan	√	
21.6	2,3,6-tri-O-methyl-d-galactopyranose	√	√
21.8	2-O-methyl-D-mannopyranosa		√
25.5-28.0	1,6-anhydro-β-D-glucopyranose		√
25.8-30.0		√	

面粉糨糊原料成本低、易获取,糨糊使用后能增强纸张的机械强度且对书画外观没有明显影响,而且糨糊作为黏结材料其操作具备可逆性,通过适当增加湿度即可将经糨糊黏结的纸张完全分离而不损伤纸张,因此糨糊一直是我国传统纸质书画装裱必备材料,中国古代书画装裱的专著,明代周嘉胄《装潢志》中“墨以胶成,裱以糊就”的论述即是关于糨糊的记载^[3]^。此外,面粉、糯米浆等多糖材料都是我国古代建筑以及家具等器物制作常用原材料,其中古建地仗或者器物灰胎制作中面粉经常与桐油、猪血等混合用作胶结材料^[1,2]^,而糯米浆则能增强建筑灰浆的强度、韧性和防渗能力^[28]^。

## 3 结论

Py-GC/MS技术在文物有机材料的识别及研究方面具有十分广阔的应用前景。本研究基于Py-GC/MS技术系统分析了中国古代书画、建筑、器物等文物中常用的多糖类胶结材料淀粉、桃胶,以及西方国家最常用的阿拉伯胶。研究确定了3类材料的热裂解产物,并发现其热裂解产物在保留时间前、中、后段分别呈现出明显的一致性或差异性,因此研究通过特定保留时间段内的差异性实现了淀粉、桃胶以及阿拉伯胶的辨别。

将所建立的多糖材料分析方法用于故宫旧藏清代一件漆器文物以及一件书画文物样品胶结材料的分析,在两个样品中均检测到淀粉。实验结果与相应类别文物传统用料能够相互印证,这也验证了所建立Py-GC/MS分析方法的可行性。但需要指出的是,两件文物样品中所检出的淀粉裂解产物明显少于淀粉标准样品,这与文物中淀粉的自然老化有关,也可能与文物样品中其他有机、无机材料对检测结果的影响有关,因此,多糖类材料的老化,以及其他材料对多糖裂解过程及裂解产物检测的影响都有待深入研究。
